# Psychometric Properties of the Persian Version of Sickness Impact Profile-30 (SIP-30) in Community-Dwelling Older Adults

**DOI:** 10.1155/jare/9959086

**Published:** 2025-01-06

**Authors:** Sajad Siavash, Amin Ghaffari, Ghorban Taghizadeh, Laleh Lajevardi, Akram Azad

**Affiliations:** ^1^Department of Occupational Therapy, Rehabilitation Research Center, School of Rehabilitation Sciences, Iran University of Medical Sciences, Tehran, Iran; ^2^Department of Occupational Therapy, Musculoskeletal Research Center, School of Rehabilitation Sciences, Isfahan University of Medical Sciences, Isfahan, Iran

**Keywords:** older adults, Persian, psychometrics, reliability, validity

## Abstract

**Purpose:** Accurate measurement tools are essential for evaluating the health-related quality of life in older adults. We aimed to translate and evaluate the psychometric properties of the Sickness Impact Profile-30 (SIP-30) in community-dwelling older adults.

**Materials and Methods:** One hundred and fifty older adults participated in this study. To evaluate construct validity, its correlation with General Health Questionnaire-28, Geriatric Depression Scale-15, Hospital Anxiety and Depression Scale, Modified Health Assessment Questionnaire, Numeric Pain Rating Scale, and Fullerton Advanced Balance Scale was assessed. Reliability features were also investigated.

**Results:** The results of construct validity analysis demonstrated a moderate to high (*r* = 0.61–0.84) correlation between the total score of SIP-30 and GHQ-28, GDS-15, HADS, MHAQ, and NPRS. There was a moderate inverse (*r* = −0.67) correlation between the total score of the SIP-30 and the FAB Scale. Test–retest reliability (ICC > 0.83) and internal consistency (*α* = 0.94) of the Persian SIP-30 were high.

**Conclusions:** The results indicated that the Persian SIP-30 is a reliable and valid measure to assess health-related quality of life in community-dwelling older adults.

## 1. Introduction

Aging is a multifaceted process defined by the gradual accumulation of cellular damage, a progressive decline in function, and an increased susceptibility to a range of diseases. It is intricately intertwined with the onset and progression of age-related conditions, including cardiovascular and neurodegenerative diseases [1]. The number of individuals aged 60 years and older is forecasted to reach 2.1 billion by 2050 [2]. As people age, their functional abilities decline and impact their health-related quality of life (HRQOL) with increased healthcare costs [3]. HRQOL serves as a valuable assessment tool in the context of treatment, particularly among elderly individuals, where enhancing physical and mental capabilities after they have diminished can be challenging [2].

HRQOL measures disease and treatment outcomes from the patient's perspective, offering insights into an individual's overall well-being by assessing their physical health and mental health and how these factors impact their quality of life [4, 5]. Detailed information regarding HRQOL provided through valid and reliable tools allows comparisons among various populations.

The Sickness Impact Profile (SIP) was developed in the 1970s by researchers at the University of Washington, led by Barbara Bergner, to assess the impact of illness on quality of life. SIP among the widely used measures that have been used in previous studies for evaluating HRQOL and health-related functional status can be evaluated using the SIP as a generic measure [6]. Its broad acceptance is primarily due to its clinical thoroughness and focus on observable behaviors, unlike other quality-of-life measures such as the Nottingham Health Profile or the SF-36, which rely more on subjective health perceptions [7]. This measure can apply to a wide range of disorders with diverse severities and cultural heritages. Psychometric features of the SIP have been studied in several communities [8].

The original SIP comprises 136 items congregated in 12 subscales that measure how sickness affects daily activities and behavior across two physical and psychosocial dimensions. Developed through extensive literature review, patient input, and testing, it has been validated for reliability in various populations, making it a widely used tool in health status and clinical research [8]. This lengthy measure was tiring and time-consuming for respondents. Hence, van Straten et al. revised the original SIP (i.e., elimination of stroke-unrelated items and subscales and removal of items that did not elicit the consistency of the remaining subscales) and extracted 30 items from the original SIP.

This 30-item measure (SIP-30) is grouped into eight subscales including body care and movement, social interaction, mobility, communication, emotional behavior, household management, alertness behavior, and ambulation. While the SIP-30 was initially developed to measure HRQOL in populations like stroke survivors, its broader purpose is to assess the impact of health conditions on daily functioning and overall well-being across various domains, including physical, emotional, and social aspects [9]. Previous studies have shown the importance of the areas measured by the SIP-30 in older adults [10–13]. Testing its validity in community-dwelling older adults is justifiable because many validated tools, including the SIP-30, have been designed to adapt to different populations. Testing its validity in a new demographic is a common and necessary practice to confirm that it accurately captures the intended constructs in diverse groups. Furthermore, older adults, particularly those living independently in the community, may face physical, cognitive, and social challenges that are similar to those experienced by stroke survivors. Therefore, validating the SIP-30 in this population ensures it is a robust and applicable measure for assessing HRQOL in different contexts. This study aimed to translate and examine the psychometric attributes of the Persian SIP-30 in community-dwelling older adults.

## 2. Methods

### 2.1. Study Design and Participants

In this cross-sectional and analytical study, elderly people living in the community were recruited through a straightforward nonrandomized method between March 2022 and June 2023, in Tehran, Iran. These individuals live independently in the community and actively engage in occupations such as shopping and administrative work. Additionally, these elderly people experience the normal conditions associated with aging. Participants were included if (a) they were aged 60 years and over; (b) being Iranian and living in Iran for at least 5 years; (c) being literate in Persian language; (d) having the ability for verbal communication; and (e) lack of cognitive decline (Mini-Mental Scale Examination score ≥ 21) [14]. Participants were excluded if they failed to attend a retest on time (i.e., two weeks later).

Before study participation, ethical approval was given by the Ethical Committee of Iran University of Medical Sciences (IUMS) (IR.IUMS.REC.1400.1240). Written informed consent was obtained from all individuals. Data collection was conducted in a quiet room between 9:00 a.m. and 1:00 p.m. at the school of rehabilitation sciences, IUMS in Tehran City. Permission for translation and examining psychometric properties of the SIP-30 in community-dwelling older adults was obtained from the SIP-30 developer, Prof. Annemieke van Straten.

### 2.2. Procedures

The translation process and linguistic adjustment were commenced after obtaining authorization from Prof. Annemieke van Straten to conduct this research in the Persian language based on the International Quality of Life Assessment protocol [15]. First, forward (i.e., original English into Persian) translation was carried out by two fluent Persian translators independently. These two initial Persian translations were assessed conceptually, idiomatically, semantically, and culturally by an expert panel of occupational therapists. Then, two different native English speakers performed the backward (i.e., Persian into English) translation. The translators and authors organized an expert committee and evaluated these two versions of the backward translations (Table A1). The final version of this profile was made following this session. During the translation process, some items were modified based on participants' understanding of the concepts. Items #8, #25, and #28 were changed from “I show less affection,” “I am confused and start several actions at a time,” and “I don't walk up or down hills” to “I show less affection from myself,” “I am impatient and start several actions at a time,” and “I don't walk inclines and declines,” respectively.

### 2.3. Validity

*Face validity* was examined by calculating the impact score based on the opinions of 20 community-dwelling older adults. Their task was to give their opinion on the items' ambiguity and comprehensibility.

*Content validity* was evaluated based on the Lawshe method with 10 experts (i.e., occupational therapists and physical therapists) working in geriatrics rehabilitation. The experts assessed the suitability, quality, and quantity of the SIP-30 items. The values of content validity ratio (CVR) and content validity index (CVI) were quantified [16].

*Construct validity* was assessed by exploring the relationship between the SIP-30 as a tool designed to assess HRQOL across various dimensions, including physical and psychosocial dimensions and General Health Questionnaire-28 (GHQ-28), Geriatric Depression Scale-15 (GDS-15), Hospital Anxiety and Depression Scale (HADS) alongside the psychosocial dimension of SIP-30 and Modified Health Assessment Questionnaire (MHAQ), Numeric Pain Rating Scale (NPRS), and Fullerton Advanced Balance (FAB) Scale related to the physical dimension of SIP-30 by using Pearson correlation coefficient.

*Discriminant validity* was determined by measuring the SIP-30's ability to distinguish between two groups (with and without fall risk) of older adults based on FAB Scale scores.

### 2.4. Reliability

*Test–retest reliability* was assessed by calculating the correlation between test scores acquired from initial and second evaluations, conducted with a 2-week time interval [17].

### 2.5. Measurements

The SIP-30 is a modified version of the SIP devised for individuals affected by a stroke. It consists of 30 items that are divided into two dimensions: physical and psychosocial. The physical dimension includes body care and movement (Items 1–5), mobility (Items 11–13), household management (items 21–24), and ambulation (Items 28–30). On the other hand, the psychosocial dimension includes social interaction (Items 6–10), communication (Items 14–16), emotional behavior (Items 17–20), and alertness behavior (Items 25–27). Each item is answered dichotomously (Yes = 1/No = 1), resulting in a total score of 30. Lower scores reflect better quality of life [18, 19].

The GHQ-28 is a tool for the identification of people who are probable to have or are at risk of developing psychiatric disorders. This questionnaire consists of 28 items that measure emotional distress with 4 somatic symptoms, anxiety or insomnia, social dysfunction, and severe depression subscales. The Persian GHQ-28 has sufficient validity and reliability (Cronbach's *α* = 0.94) in community-dwelling older adults [20].

The GDS-15 is a screening scale for evaluating clinical depression among older adults. Each item is rated 0 (No) or 1 (Yes), yielding a total score between 0 and 15. On this scale, a score higher than five indicates depression. The test takes between 5 and 7 min and can be completed by self-report or by the examiner. The Persian GDS-15 has appropriate validity and reliability (Cronbach's *α*: 0.9) in community-dwelling older adults [21].

The HADS is a self-report screening tool that was initially devised to measure anxiety and depression states in outpatient individuals. This scale consists of 2 subscales with seven items. The rating of each item ranges from 0 to 3, with scores ranging from 0 to 21 for each subscale. This scale has acceptable reliability and validity attributes in the Persian language and older adults [22, 23].

The MHAQ was developed to measure functional capacity while carrying out daily chores. The MHAQ consists of eight items derived from each HAQ subscale. The total score is between 0 and 3. The scores of each of the answers are as follows: no problem (0 points), with a little difficulty (1 point), with a lot of difficulty (2 points), and I can't (3 points). Higher scores indicate worse performance and greater disability. Scores less than 0.3 are considered normal. It is suggested that the scores be divided into mild (MHAQ < 1.3), moderate (1.3 < MHAQ < 1.8), and severe (MHAQ > 1.8) disability categories. Administration takes less than 5 minutes [24]. The Persian MHAQ has been studied in older adults residing in nursing homes (Cronbach's *α*: 0.98) [25].

The NPRS is a subjective scale in that individuals evaluate their pain level on a numerical scale from 0 (*no pain at all*) to 10 (*worst imaginable pain*). The test–retest reliability of this scale has been reported to range from 0.67 to 0.96. Additionally, the convergence validity of this test with the eye scoring scale has been reported to range from 0.79 to 0.95 [26].

The FAB Scale is designed to identify various elements contributing to balance. It evaluates sensory, musculoskeletal, and neuromuscular systems that contribute to balance. This scale contains 10 items scored on a 5-point rating scale. The maximum score is 40. A score of ≤ 25 denotes the risk of falling. The Persian FAB Scale has been validated (Cronbach's *α*: 0.84) in community-dwelling older adults [27].

### 2.6. Statistical Analysis

All statistical analysis was executed using IBM SPSS, Version 20.0. The Kolmogorov–Smirnov test was utilized to verify normal distribution. Descriptive statistics (mean, standard deviation [SD], and frequency) were used for demographic characteristics. Face validity was determined by calculating the item impact score for each item with a five-point Likert scale. An item score ≥ 1.5 was considered acceptable for each item [28]. For establishing content validity, CVR and CVI values were calculated. According to the Lawshe method, CVR values > 0.62 were considered acceptable. The CVI values ≥ 0.79, 0.79–0.70, and < 0.70 were deemed appropriate, questionable, and unacceptable, respectively [16]. The construct validity was explored by examining the relationship between the total SIP-30 score and GHQ-28, GDS-15, HADS subscale scores, MHAQ, NPRS, and FAB Scale scores using Pearson correlation coefficient (high = 0.89–0.70; moderate = 0.69–0.50) [29]. The independent *t*-test was used to examine discriminant validity in distinguishing older adults with and without fall risk based on FAB Scale scores (score of ≤ 25). Test–retest reliability was calculated by using an intraclass correlation coefficient (ICC), with a 95% confidence interval. ICC values were interpreted as follows: > 0.80, 0.8–0.6, and < 0.6 indicate high, moderate, and low reliability, respectively. Additionally, the kappa coefficient was calculated for each item of the SIP-30, which was interpreted as slight: 0.01–0.20, fair: 0.21–0.40, moderate: 0.41–0.60, substantial: 0.61–0.80, and almost perfect: 0.81–1. The standard error of measurement (SEM) and minimal detectable change (MDC) were calculated as $\text{SEM}={\text{SD}}_{\text{pooled}}\times \sqrt{1-\text{ICC}}$ and ${\text{MDC}}_{95\%}=\text{SEM}\times \sqrt{2}\times 1.96$. An SEM value < ½ SD was considered acceptable [30–32]. Internal consistency of the SIP-30 was determined using Cronbach's *α* coefficient, acceptable at > 0.7 [33]. Factor analysis was performed to check that each of the SIP-30 test items is related to the physical or psychosocial dimensions along with Cronbach's *α* coefficient with item deletion. Ceiling and floor effects were estimated by calculating the proportion of respondents obtaining the maximum and minimum possible score (criterion < 20%), respectively [34]. The significance level was set as *p* < 0.05.

## 3. Results

One hundred and fifty older adults (76 men and 74 women) participated in this study with a mean ± SD age of 71.34 ± 7.24 years. The mean ± SD total score of the SIP-30 was 9.07 ± 8.16, ranging from 0 to 28. The demographic characteristics of the sample are shown in Table 1.

Item impact scores ranged from 2.8 to 4.18 for all items. The CVR (0.96–1.0) and CVI (0.8–1.0) values were in the acceptable range. The results of the construct validity analysis demonstrated a significant positive moderate to high correlation (*r* = 0.61–0.84; *p* < 0.05) between the total score of SIP-30 and GHQ-28, GDS-15, HADS subscale scores, MHAQ, and NPRS. However, there was an inverse moderate (*r* = −0.67; *p* < 0.05) correlation between the total SIP-30 score and the FAB Scale (Table 2). Regarding discriminant validity, there was a significant (*t* = 115.05; *p* < 0.05) difference between older adults with and without fall risk (Table 3).

As shown in Table 4, the ICCs for the subscale and total score of the SIP-30 were from 0.83 to 0.99 and 0.98, respectively. The range of kappa agreement for each item of the SIP-30 was 0.63–1.00 (mean = 0.88), implying substantial to almost perfect agreement. The SEM was in the acceptable range for the total (SEM = 1.14) and subscale (0.2–0.51) scores of the SIP-30. The MDC values were from 0.42 to 1.41 for subscale scores and 3.16 for the total score of the SIP-30.

Factor analysis of SIP-30 was performed using principal component analysis (PCA). After Varimax rotation, the rotated sums of squared loading revealed a two-factor structure (eigenvalue > 1), with the first factor explaining 37.5% and the second factor explaining 7.46% of the variance, together accounting for 44.96% of the variance. Factor 1 includes Items 1, 2, 3, 4, 5, 10, 11, 12, 13, 14, 16, 21, 22, 23, 24, 27, 28, 29, and 30 and Factor 2 included items 6, 7, 8, 15, 17, 18, 19, and 26. Also, Items 9 and 20 were equal in both factors. Cronbach's *α* was 0.94 for all items of the SIP-30. “Social interaction” (*α* = 0.68), “communication” (*α* = 0.46), and “alertness behavior” (*α* = 0.58) did not reach satisfactory Cronbach's *α* coefficient value. Cronbach's *α* after deletion of each item remained consistent (0.94). The range of correlation between the SIP-30 items is between 0.9 and 1 (Table 5).

The ceiling and floor effects for the total SIP-30 score (ceiling: 0%; floor: 16%) and its subscales were as follows, respectively: body care and movement (3.3%, 59.3%), social interaction (5.3% and 30.7%), mobility (26.7% and 34%), communication (26.7% and 37.3%), emotional behavior (8% and 58%), household management (14% and 54.7%), alertness behavior (12.7% and 40.7%), and ambulation (15.3% and 52%).

## 4. Discussion

This is the first psychometric study of the SIP-30 among older adults who reside in a community. Considering the target population of this study, the scale items should be comprehensible. Therefore, translation and adjustment of this scale are of great importance. The results showed that the Persian SIP-30 has acceptable face validity, content validity, construct validity, discriminant validity, test–retest reliability, and internal consistency.

Content validity implies that an instrument covers all aspects or dimensions related to the concept being measured [35]. This is an important element in the development of clinical instruments, as it ensures that the scale accurately quantifies the proposed construct [36]. The content validity of the Persian SIP-30 was found to be appropriate in terms of CVR and CVI values. These quantitative data can be practical for health professionals in the target culture.

The extent to which an instrument accurately reflects the underlying theoretical construct it was designed to measure is known as construct validity [37]. Construct validity is important for ensuring that therapists use valid measures in clinical settings [38]. The results showed a significant moderate to high correlation between the SIP-30 and GHQ-28, GDS, HADS subscales, MHAQ, NPRS, and FAB Scale.

There was a high correlation between the SIP-30 and GHQ-28. Both SIP-30 and GHQ-28 are standard self-report measures that evaluate HRQOL and psychological distress in individuals. Specifically, the SIP-30 is a multidimensional scale that assesses the impact of health conditions on individuals' physical and psychosocial functioning, including activities of daily living, mobility, communication, social interaction, and emotional well-being. Similarly, the GHQ-28 is a widely used measure of psychological distress that assesses a person's current state of mental health, including symptoms of anxiety, depression, somatic complaints, and social dysfunction. Furthermore, both measures are relatively brief and easy to administer, making them practical tools for clinical and research settings.

Additionally, SIP-30 showed a high correlation with GDS and HADS depression subscale. Depression is a mental illness that not only affects a person's mental health but also has physical health consequences. One of the mechanisms by which depression affects physical health is how it affects the immune system. Literature has shown that depression can lead to dysregulation of the immune system, resulting in chronic inflammation, which is associated with numerous health problems, such as cardiovascular diseases, diabetes, and cancer [39–41]. Furthermore, individuals with depression have an imbalance in occupational performance and experience reduced health related to quality of life due to limited participation in daily activities [42–44]. Therefore, addressing both the mental and physical health components of depression is essential for achieving comprehensive health outcomes. Based on this depressed people have a higher score on the SIP-30 test and clinicians should recognize depression as a key factor affecting HRQOL in their assessments and interventions.

Furthermore, the results indicated a high correlation between the SIP-30 and MHAQ disability level questionnaire. Poor health is associated with disability through various mechanisms. Chronic diseases like diabetes, cardiovascular disease, and arthritis can lead to limitations in mobility and performing daily activities and social participation restrictions [45]. Additionally, mental health issues (e.g., anxiety and depression) can result in cognitive, emotional, and physical impairments [46]. These mechanisms highlight the importance of addressing physical and mental health needs for the prevention and management of disability in older adults who reside in a community.

Moreover, the results showed a moderate correlation between the SIP-30 and the HADS anxiety subscale and NPRS. Anxiety and chronic pain can lead to excessive activation of the hypothalamus–pituitary–adrenal axis and increased secretion of cortisol (i.e., stress hormone) that ultimately leads to health problems such as pathological increase in heart rate and blood pressure [47, 48]. Controlling blood pressure can prevent heart disease and strokes in the elderly [49]. Therefore, therapists should also consider managing anxiety in older adults. Besides, chronic pain can result in immune system dysfunction, increased infection, and related diseases (e.g., diabetes, arthritis, and cardiovascular diseases) through excessive activation of the sympathetic nervous system [50, 51]. Thus, pain in the elderly should be considered as one of the health-related factors related to quality of life.

The results showed a moderate and inverse correlation between the SIP-30 and the FAB Scale. The ability to maintain balance depends on several physiological systems, including visual, vestibular, and proprioceptive systems [52]. Disorders of any of these systems can lead to impaired balance and increased fall risk, which can lead to injuries [53]. Verghese et al. stated that poor balance is a significant predictor of mortality among community-dwelling older adults. They also concluded that balance scales can serve as useful screening tools for recognizing individuals who are in danger of adverse health outcomes [54].

Discriminant validity is the ability of a measure to distinguish between groups that are expected to differ based on a known characteristic or diagnosis [55]. We found a significant difference in the total SIP-30 score of older adults with and without fall risk grouped based on FAB Scale scores. Fall risk is a key element in determining older adults' quality of life [56]. This result is backed by an earlier study that indicated that a person's perceived quality of life is a vital component in foreseeing fall risk.

Internal consistency refers to the amount items of a scale are related or associated and, therefore, measure the same underlying construct [57]. Acceptable internal consistency was obtained for the total score and body care and movement, mobility, emotional behavior, household management, and ambulation subscales (Cronbach's *α* = 0.72–0.94). These results were similar to the results of van Straten et al. and Carod-Artal et al. [9, 19], except for the ambulation subscale. This subscale was not considered acceptable in the aforementioned studies. Internal consistency for three (social interaction, communication, and alertness behavior) subscales did not reach an acceptable level (Cronbach's *α*: 0.46–0.68). These results were in line with previous studies [9, 19], except for the alertness behavior subscale, which was reported to have acceptable internal consistency by van Straten et al. [9]. Several factors can contribute to an increase in Cronbach's *α*, including high number of items, homogeneity of items, high variance of items, high interitem correlation, scale format (continuous scoring leads to higher Cronbach's *α*), high sample size, and low heterogeneity of the sample [33, 58]. The value of Cronbach's *α* is reliant on the number of items residing in a subscale. Low Cronbach's *α* in social interaction, communication, and alertness behavior subscales could be attributed to their limited number of items (i.e., 3 to 5 items). There are no specified item numbers that guarantee a high Cronbach's *α*, since it depends on various factors (e.g., complexity of the construct being measured, heterogeneity of items, and sample size). However, a commonly cited rule of thumb is to aim for at least 5–10 items in a subscale [58]. Since Cronbach's *α* did not change after the deletion of each item, it can be concluded that the total score, subscale scores, and individual items of this scale are clinically acceptable for assessing HRQOL.

The results showed high test–retest reliability for the total and subscale scores of the Persian SIP-30, which were consistent with Carod-Artal et al.'s study on stroke survivors [19]. Furthermore, the kappa coefficient in the test–retest for all items of the Persian SIP-30 was substantial to almost perfect. Possible reasons for these results could be clear item phrasing, straightforward administration and scoring instructions, and an appropriate retesting interval. The MDC results showed that changes greater than 3.16 are not caused by measurement error and represent a real improvement with 95% confidence in clinical practice.

The Persian SIP-30's factor analysis findings are partially consistent with the original version, which identified a two-factor structure for the SA-SIP30, distinguishing between physical and psychosocial dimensions [9]. The accumulation of scores at the upper (ceiling) or lower (floor) end of the scale is reported by the ceiling and floor effect [59]. There was no ceiling or floor effect for the total score of the Persian SIP-30. However, the ceiling and floor effects for subscales ranged from 3.3% to 26.7% and 30.7% to 59.3%, respectively. The result of the ceiling effect was similar to the study of Artal et al. in stroke survivors. A higher ceiling effect in the current study contrasted to Carod-Artal et al.'s study could be because participants (i.e., community-dwelling older adults) have fewer HRQOL problems compared to stroke survivors [19]. Therefore, a higher percentage of scores were accumulated at the lower end of the score range. The results show that the total score of the SIP-30 does not have a ceiling effect, but some of the subscales do. Finally, the Persian SIP-30 as a multidimensional measure demonstrates acceptable validity and reliability and can effectively show changes in improving the HRQOL in community-dwelling elderly people.

## 5. Conclusion

The results suggested that the Persian SIP-30 is a valid and reliable scale for assessing HRQOL in community-dwelling older adults. The Persian SIP-30 can be used in routine clinical practice and research studies. This study expands the existing evidence regarding the SIP-30 psychometrics.

### 5.1. Study Limitations

The current study may have some limitations. This study's implementation period was during the COVID-19 pandemic, which caused less access to the elderly living in the community, so we used convenience sampling. Future studies should consider employing probability sampling methods. Another limitation is that only older adults with acceptable cognitive function (Mini-Mental Scale Examination score ≥ 21) were recruited. To ensure the generalizability of our findings, we need to take these limitations into account and address them in future studies.

## Figures and Tables

**Table 1:** Demographic and clinical data of the study sample (*N* = 150).

Characteristics	Number (%)/mean ± SD (range)
Age (y)	71.34 ± 7.24 (60–90)
Gender	
Male/female	76 (50.7%)/74 (49.3%)
Marital status	
Single/married/divorced or widowed	1 (0.75%)/106 (70.7%)/43 (28.6%)
Educational level	
High school or less/university degree	105 (70%)/45 (305)
Living arrangements	
Alone/with spouse or relatives	40 (26.7%)/110 (73.3%)
Medical conditions	
Cardiovascular/metabolic/others/multiple comorbidities/none	20 (13.3%)/18 (12%)/20 (13.3%)/44 (29.3%)/48 (32%)
Medication usage	
Yes/no	58 (38.7%)/92 (61.3%)
Assistive device use	
Yes/no	32 (21.3%)/118 (78.7%)
Fall history	
Yes/no	43 (28.7%)/107 (71.3%)
Fall risk (based on FAB Scale scores)	
Yes (26–40)/no (0–25)	43 (28.7%)/107 (71.3%)
Sickness Impact Profile-30	
Total score/body care and movement/social interaction/mobility/communication/emotional behavior/household management/alertness behavior/ambulation	9.07 ± 8.16 (0–28)/1.03 ± 1.52 (0–5)/1.61 ± 1.52 (0–5) 1.39 ± 1.21 (0–3)/1 ± 0.96 (0–3)/0.94 ± 1.32 (0–4)/1.14 ± 1.49 (0–4)/1.03 ± 1.05 (0–3)/0.94 ± 1.14 (0–3)
General Health Questionnaire-28	22.89 ± 14.02 (3–68)
Geriatric Depression Scale-15	4.10 ± 3.78 (0–15)
Hospital Anxiety and Depression Scale	
Anxiety subscale	5.54 ± 3.71 (0–15)
Depression subscale	6.89 ± 4.39 (0–19)
Modified Health Assessment Questionnaire	0.44 ± 0.56 (0–2.13)
Numeric Pain Rating Scale	3.39 ± 2.40 (0–9)
Fullerton Advanced Balance Scale	28.67 ± 8.55 (1–39)

Abbreviations: FAB Scale = Fullerton Advanced Balance Scale, SD = standard deviation.

**Table 2:** Correlation between the SIP-30 and its subscales with other health-related scales (*N* = 150).

Variables	GHQ-28	GDS-15	HADS		MHAQ	NPRS	FAB Scale
			Anxiety	Depression			
Sickness Impact Profile-30 total score	0.81	0.81	0.67	0.72	0.84	0.61	−0.67
Body care and movement subscale	0.72	0.61	0.52	0.58	0.86	0.64	−0.70
Social interaction subscale	0.67	0.72	0.60	0.65	0.56	0.34	−0.38
Mobility subscale	0.53	0.56	0.43	0.45	0.67	0.52	−0.54
Communication subscale	0.53	0.60	0.48	0.50	0.55	0.33	−0.47
Emotional behavior subscale	0.81	0.82	0.75	0.73	0.65	0.55	−0.42
Household management subscale	0.67	0.63	0.49	0.59	0.74	0.46	−0.63
Alertness behavior subscale	0.62	0.69	0.59	0.58	0.56	0.45	−0.41
Ambulation subscale	0.55	0.49	0.44	0.45	0.71	0.53	−0.64

*Note:* All correlations were significant at *p* < 0.05.

Abbreviations: FAB = Fullerton Advanced Balance, GDS-15 = Geriatric Depression Scale-15, GHQ-28 = General Health Questionnaire-28, HADS = Hospital Anxiety and Depression Scale, MHAQ = Modified Health Assessment Questionnaire, NPRS = Numeric Pain Rating Scale.

**Table 3:** Discriminant validity of Persian Sickness Impact Profile-30 (*N* = 150).

Variables	Without fall risk	With fall risk	*t*
	Mean ± SD	Mean ± SD	
SIP-30 total score	5.66 ± 5.66	17.56 ± 8.19	115.05
Body care and movement	0.41 ± 0.90	2.56 ± 1.66	103.33
Social interaction	1.22 ± 1.26	2.60 ± 1.66	30.83
Mobility	0.94 ± 1.09	2.49 ± 0.67	74.97
Communication	0.70 ± 0.77	1.77 ± 0.95	52.29
Emotional behavior	0.58 ± 1.06	1.84 ± 1.49	33.89
Household management	0.61 ± 1.04	2.47 ± 1.61	69.87
Alertness behavior	0.73 ± 0.86	1.77 ± 1.11	37.42
Ambulation	0.50 ± 0.87	2.02 ± 0.99	86.01

*Note:* All statistical analyses were significant at *p* < 0.05.

**Table 4:** The reliability results of the Persian Sickness Impact Profile-30 (*N* = 150).

Variables	Intraclass correlation (95% confidence interval)	Standard error of measurement	Minimal detectable change	Internal consistency
Sickness Impact Profile-30 total score	0.98 (0.97–0.99)	1.14	3.16	0.94
Body care and movement	0.99 (0.98–0.99)	0.15	0.42	0.83
Social interaction	0.88 (0.83–0.91)	0.51	1.41	0.68
Mobility	0.94 (0.91–0.95)	0.30	0.83	0.72
Communication	0.92 (0.89–0.94)	0.27	0.75	0.46
Emotional behavior	0.94 (0.92–0.96)	0.32	0.89	0.78
Household management	0.98 (0.98–0.99)	0.21	0.58	0.85
Alertness behavior	0.83 (0.78–0.88)	0.43	1.19	0.58
Ambulation	0.97 (0.95–0.98)	0.20	0.55	0.76

**Table 5:** Factor analysis of the Persian Sickness Impact Profile-30 (*N* = 150).

Items	Components		Correlation with total score	Cronbach's *α* coefficient with item deletion	Cronbach's *α* coefficients
	Factor 1	Factor 2			
1	0.753	0.147	0.65	0.94	0.94
2	0.672	0.299	0.68	0.94	
3	0.616	0.355	0.67	0.94	
4	0.727	0.277	0.71	0.94	
5	0.490	0.092	0.41	0.94	
6	0.196	0.631	0.49	0.94	
7	0.169	0.666	0.49	0.94	
8	0.101	0.654	0.43	0.94	
9	0.401	0.391	0.53	0.94	
10	0.419	0.300	0.49	0.94	
11	0.501	0.327	0.57	0.94	
12	0.563	0.123	0.50	0.94	
13	0.634	0.222	0.61	0.94	
14	0.624	0.156	0.56	0.94	
15	0.084	0.502	0.34	0.94	
16	0.450	0.310	0.51	0.94	
17	0.444	0.635	0.69	0.94	
18	0.217	0.637	0.51	0.94	
19	0.244	0.623	0.53	0.94	
20	0.466	0.509	0.63	0.94	
21	0.547	0.283	0.57	0.94	
22	0.676	0.293	0.68	0.94	
23	0.635	0.367	0.70	0.94	
24	0.622	0.438	0.72	0.94	
25	0.001	0.598	0.32	0.94	
26	0.370	0.580	0.61	0.94	
27	0.630	0.348	0.69	0.94	
28	0.778	0.038	0.56	0.94	
29	0.697	0.126	0.60	0.94	
30	0.706	0.072	0.57	0.94	

*Note:* Kaiser–Meyer–Olkin (KMO): 0.83; Bartlett's test of sphericity: 2783.99. All statistical analyses were significant at *p* < 0.05.

**Table 6:** Backward translation of Persian Sickness Impact Profile-30.

No	Subscales	Items
1	Body care and movement	I make difficult moves with the help of others (for example, getting in and out of cars, bathtubs)
2		I move my hands or fingers with restriction or difficulty
3		I get in and out of the bed or chair by holding some things for support or using a cane or walker
4		It is difficult for me to put on shoes, socks or stockings
5		I can only get dressed with someone's help

6	Social interaction	I show less interest in other people's problems; for example, I do not listen when they talk about their problems and I do not offer to help
7		I often deal with people around me with a bad temper and anger, for example, I speak badly to them, I give sharp and stinging replies, and I easily criticize
8		I show less affection
9		I rarely do group and social activities with others
10		I talk less to people around me

11	Mobility	I stay home most of the time
12		I am not going downtown
13		I do not go out in the dark or in dark places without the help of others

14	Communication	I can only continue the conversation if I am close to the other party or if I look at their face
15		It is difficult for me to speak, for example, I get stuck, I stutter, I mumble and slur my words
16		When I am stressed, I cannot speak clearly

17	Emotional behavior	I tell myself how bad and useless I am, for example, that I am a burden on others
18		I suddenly laugh or cry
19		I act nervously and impatiently towards myself, for example I talk badly about myself, I insult myself, I blame myself for what happens
20		I get sudden fears

21	Household management	I do not do the maintenance and repair work that I used to do in home or yard any longer
22		I do not do any of the shopping-related things I used to do
23		I do not do any of the house cleaning that I used to do
24		I do not do any of the clothes washing that I used to do

25	Alertness behavior	I am disorganized and start multiple task at a time
26		I make more mistakes than usual
27		It is difficult for me to do activities that require concentration and thinking

28	Ambulation	I do not walk uphill or downhill
29		I only get around by using a walker, crutches, cane, with the help of walls or furniture
30		I walk very slowly

## Data Availability

All data used to support the results of this study are included in the article.
